# A DNA-based method for distinction of fly artifacts from human bloodstains

**DOI:** 10.1007/s00414-021-02643-7

**Published:** 2021-06-30

**Authors:** Carla Bini, Arianna Giorgetti, Alessandra Iuvaro, Elena Giovannini, Denise Gianfreda, Guido Pelletti, Susi Pelotti

**Affiliations:** Department of Medical and Surgical Sciences, Section of Legal Medicine, University of Bologna, via Irnerio, 49, 40126 Bologna, Italy

**Keywords:** Forensic genetics, Fly artifacts, Bloodstain pattern analysis, mtDNA, COI sequence, Species identification

## Abstract

Fly artifacts resulting from insect activity could act as confounding factors on a crime scene and interfere with bloodstain pattern analysis interpretation. Several techniques have been proposed to distinguish fly artifacts from human bloodstains based on morphological approach and immunological assay, but a DNA-based method has not been developed so far. Even if in forensic genetic investigations the detection of human DNA is generally the primary goal, fly artifacts can provide useful information on the dynamics of crime events. The present study provides a molecular method to detect fly DNA from artifacts deposited by *Calliphora vomitoria* after feeding on human blood through the analysis of the mitochondrial cytochrome oxidase gene subunit I (*COI*). Fly artifacts originated from digestive process and of different morphology spanning from red and brownish/light brown, circular and elliptical stains to artifacts with sperm-like tail or a tear-shaped body were collected. The COI amplification was successfully obtained in 94% of fly artifact samples. The method showed high sensitivity and reproducibility, and no human DNA contamination was observed, offering specificity for use in confirmatory test. This molecular approach permits the distinction of fly artifacts from genuine bloodstains and the identification of fly’s species through the COI region sequencing by protocols usually applied in forensic genetic laboratories.

**Supplementary Information:**

The online version contains supplementary material available at 10.1007/s00414-021-02643-7.

## Introduction

In violent deaths, bloodstain pattern analysis (BPA) can provide useful information about the physical events that led to bloodstains deposition, such as the nature of weapon used, the type of injury, and the approximate positions of the individuals and objects in space [1]. However, confusion in the BPA interpretation can arise because bloodstains can be altered after their formation. Some alterations could be considered as part of the bloodstain pattern and used to recreate the death scene [2], but others can act as confounding factors. This is the case of insect stains defined as “bloodstains resulting from insect activity” by the Scientific Working Group on Bloodstain Pattern Analysis (SWGSTAIN) [3]. Fecal, regurgitant, and insect-mediated transfer stains are known as artifacts, and when spots or specks of transfer patterns produced by flies are morphologically similar to bloodstains, the reconstruction of events at crime scene can be compromised [4]. Moreover, as blowflies feed on and digest biological fluids as blood, semen, and saliva, fly artifacts could contain sufficient amounts of human biological material to generate full or partial DNA profiles of the donor [5].

In previous years, several techniques have been used to differentiate fly artifacts from human bloodstains. Some authors initially proposed morphological approaches, resulting in general rules [6] or analytical flow charts [7], but due to the wide range of fly artifacts, it was impossible to provide a definition or universal catalog of their morphology. Both visual and contextual analyses have resulted often to be inconclusive, and have been reported to mostly rely on the experience and opinion of the analyst rather than on standardized and reproducible methodology [8]. In particular, when isolated artifacts are not part of a pattern, a confirmatory test to distinguish them from genuine bloodstains may avoid misidentification and false reconstruction of the crime event.

As reported in Pelletti et al. [9], Scanning Electron Microscopy (SEM) allows the visualization of morphological differences between fly artifacts deposited by *Sarcophaga carnaria* and blood controls, resulting in a suitable tool to perform a qualitative differential diagnosis between fly artifacts and bloodstain under experimental condition.

In the last years, Rivers et al. [10, 11] tested a polyclonal antiserum (anti-md3 serum) generated toward a unique cathepsin D proteinase that has been shown to react with regurgitate and defecatory fly artifacts produced by different species of blowflies, but not with transfer patterns or blood controls from humans or other animals. Recently, the study was extended to other stains produced by different species of flies following the consumption of semen, saliva, feces, and urine, showing that more than 94% of fly artifacts reacted positively with anti-md3 [12].

So far, no molecular approach has been proposed to discriminate fly artifacts from genuine bloodstains. Considering that fly artifacts originate from a mixture of human and fly biological material and that the cytochrome *c* oxidase subunit I (COI) gene is the standard locus for DNA barcoding in invertebrates and for identification of forensically important fly taxa [13–15], this study aimed to develop a suitable molecular approach to assess the presence of fly’s COI gene in fly artifacts from human blood in order to distinguish them from genuine bloodstains.

## Materials and methods

### Scene setting up

Ten milliliters of fresh human blood from a male volunteer were placed by a 3-ml plastic pipette on the floor of a scaled-down room analog, referred to herein after as fly box, and were used as blood reservoir for blowflies. The fly box was 0.12 m^3^ (1 × 0.3 × 0.4 m) with five wooden walls, and one glass wall to allow observation and for fly artifact collection. Adults of *Calliphora vomitoria* (*C. vomitoria*) were inserted in the fly box, and they were free to feed ad libitum. After 72 h, the fly box was opened for flies’ removal, and fly artifacts were collected during the following 60 days.

### Reference and fly artifact sampling

Human blood of the male volunteer was sampled as reference for DNA profiling, and pupae from *C. vomitoria* were collected as fly DNA reference.

A total of 68 fly artifact spots ranging in dimension from 2 to 7 mm and with different morphological features, except for small round and asymmetrical linear stains likely deposited by tarsi or abdomen, were sampled by swabbing with 4N6FLOQSwabs™ (Copan) from the glass wall surface of the box. All collected samples were stored at – 20 °C until DNA extraction.

### DNA extraction

After removing the puparium, DNA was extracted from the inner soft tissues of the pupae using the extraction method as described in Lehmann et al. [16] and following the manufacturer’s supplementary protocol “Purification of total DNA from insects using a disposable microtube pestle” of the DNeasy tissue kit (Qiagen). The DNA was eluted in 50 µl of Buffer AE.

The DNA from the volunteer was extracted from 10 µl of blood using QIAamp® DNA Investigator Kit (Qiagen), following the manufacturer’s protocol “Isolation of Total DNA from Small Volumes of Blood or Saliva” and eluted in 50 µl of buffer AE.

The DNA from all fly artifacts was extracted by QIAamp® DNA Investigator Kit (Qiagen) following the manufacturer’s protocols “Isolation of Total DNA from Surface and Buccal Swabs”, and the DNA was eluted in 25 µl of buffer ATE.

Negative controls were set up for all the extraction sessions.

### DNA quantitation

The Quantifiler® Trio DNA quantification kit (Applied Biosystems) was used to quantify human DNA extracts from reference blood and from a random sample of 10 fly artifacts. The quantification reactions were performed by real-time PCR (qPCR) on the Applied Biosystems 7500 Real-Time PCR System (Applied Biosystems) according to the manufacturer’s instruction.

DNA quantitation from *C. vomitoria* pupae was performed using Qubit™ dsDNA HS Assay Kit (Thermo Fisher Scientific).

### DNA amplification

A region of 440 bp of the mitochondrial cytochrome *c* oxidase subunit I (COI) gene was amplified using C1-J-1751, slightly modified, and C1-N-2191 primers previously described by Wells et al. [17]. The forward primer C1-J-1751 is modified to reduce self-dimer formation and to exclude unwanted pairing with human DNA sequences after checking primer sequences using the online software PrimerBLAST [18] and Primer Design Tools [19], as reported in Table 1.

**Table 1:** Primers sequences used in this study

	Primer	Sequence (5′-3′)	Primer length
Forward	C1-J-1751	GGATCTCCTGATATAGCTTTCCC	23
Reverse	C1-N-2191	CCCGGTAAAATTAAAATATAAACTTC	26

DNA extracts from the pupae, human blood, and fly artifact samples were used as templates for the PCR assessment. For the assessment of the method different annealing temperatures, primer concentrations and cycle number were tested. PCR reactions were performed in a final volume of 12.5 µl using 1.25 µl of GeneAmp (10 ×) PCR buffer II, 1.25 µl (25 mM) MgCl_2_, 0.6 µl of Bovine Serum Albumine (10 mg/ml), 0.25 µl (10 mM) dNTPs, 0.2 µl (5 U/µl) AmpliTaq Gold™ DNA polymerase (Applied Biosystems), 0.25 µl (10 µM) of each primer, and 2–5 µl of template DNA.

COI amplification was performed on a Veriti™ 96-Well Thermal Cycler, Thermo Fisher Scientific (Applied Biosystems), using the following cycling parameters: 10 min at 95 °C, 35 cycles for 30 s at 94 °C, 1 min at 52 °C, and 1 min at 72 °C, 5 min elongation step at 72 °C. The PCR products were electrophoresed on a GelRed stained 2% agarose gel and visualized under UV light together with a 100 base pair ladder marker.

DNAs from human blood and from the random sample of 10 fly artifacts were amplified using GlobalFiler™ IQC PCR Amplification Kit (Thermo Fisher Scientific Company, Carlsbad, USA), consisting of 21 autosomal STRs, three sex-specific markers, and two internal quality control markers to evaluate the PCR performance of the samples following the manufacturer’s instruction [20]. Positive and negative controls were set up for all sessions.

### Sequencing analysis

PCR products were purified using the ExoSAP-IT™ PCR clean-up protocol (USB Corporation) and sequenced bidirectionally using BigDye™ Terminator v1.1 Cycle Sequencing Kit (Applied Biosystems) according to the manufacturer’s protocols employing the same forward and reverse primers of PCR reaction. Sequencing was carried out on a SeqStudio Genetic Analyzer (Applied Biosystems) and analyzed by Sequencing Analysis software 7 v7.0 (Applied Biosystems). All sequences were then evaluated for species similarity on GenBank® by using the BLAST program [21, 22].

### Reproducibility, sensitivity, and mixture analyses

According to Scientific Working Group on DNA Analysis Methods (SWGDAM) guidelines, also applied for mtDNA analysis and species determination [23], the reproducibility of the method was performed on 10 random samples by three independent PCRs, and sequences were compared.

The sensitivity of the method was assessed by analysis of consecutive dilutions of total DNA extracted from *C. vomitoria* pupae. The dilutions were prepared in a range from 10 to 0.015 ng per PCR reaction.

Fly DNA at 0.2 ng was mixed with human DNA up to 20 ng in the following ratios: 20:1, 10:1, 5:1, 2:1, 1:1, 1:2, 1:5, 1:10, 1:20, 1:50, and 1:100 [24].

## Results

**Fig. 1:**
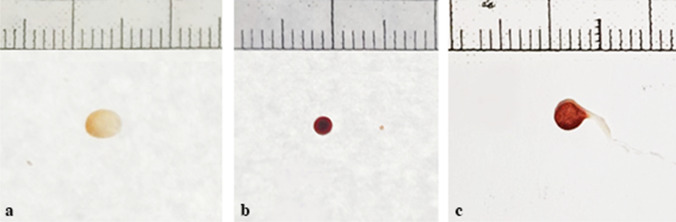
Examples of fly artifacts of different morphology submitted to the study: brownish/light brown circular or elliptical stains (**a**), red circular stains (**b**), and tailed circular or sperm-like stains (**c**)

The setting up of the fly box with *C. vomitoria* produced on all the walls several fly artifacts of different morphology spanning from red and brownish/light brown, circular and elliptical stains to artifacts with sperm-like tail or a tear-shaped body (Fig. 1).

The human DNA from the 10 quantified random fly artifacts was detected in a range from 0.002 to 2 ng/µl with degradation indexes (DI) ≤ 2. The fly DNA quantitation obtained from pupae was 56 ng/µl.

The highest PCR performance for fly’s COI gene amplification was obtained using an annealing temperature of 52 °C and varying the primer concentration up to 0.2 µM. The number of amplification cycles of 35 showed an increasing PCR fragment intensity compared to the initially tested 30 cycles, without non-specific products visualization.

**Fig. 2:**
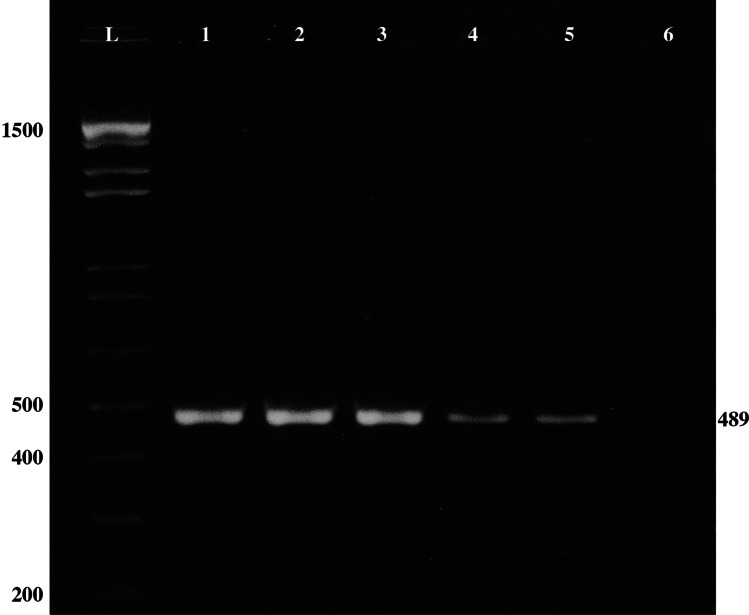
Agarose gel results of the COI region amplification of 489 bp including primers sequences. Lanes L: 100 bp DNA marker, 1: positive control (*C. vomitoria* pupae), 2–5: fly artifacts, 6: negative control (human DNA)

Following these PCR conditions, the 489 bp region of the COI gene was amplified successfully for 64 of 68 fly artifacts, of which 23/24 red circular stains, 21/24 brownish/light brown circular or elliptical stains, and all the 20/20 tailed circular or sperm-like stains (Fig. 2).

As expected, the COI primers amplified successfully the fly reference sample from pupae but failed to amplify DNA from the reference human blood sample.

The obtained sequences from different colored and shaped fly artifacts and from pupae (Online Resource 1) were subjected to the BLAST search for identification purpose and to determine their degree of similarity with respect to all multiple COI sequences present in GenBank database producing significant alignment from 99 to 100% identity with *Calliphora vomitoria* species (accession code MG969489.1) (Online Resource 2).

The three replicates of each of the 10 fly artifacts tested for reproducibility provided identical sequences. The sensitivity of the method was assessed up to an input fly DNA of 0.015 ng/µl showing a band intensity appropriate for the following sequencing analysis.

In mixture analysis, the addition of human DNA did not prevent the amplification of COI region up to fly: human DNA ratio of 1:100. Also, for sequence analysis, no difference between mixtures and pure fly DNA was found at the tested dilutions.

The DNA profiling by GlobalFiler IQC amplification kit showed full profiles from all the fly stains and without degradation and inhibition events, except for one fly artifact sample that showed a slight decrease in the electropherogram plot (data not shown). All autosomal profiles were identical and matched the human blood reference one.

## Discussion

The detection of human DNA is generally the primary goal in a forensic genetic investigation, and also insects and their activity can provide very useful information to reconstruct a crime scene or a suspected one. The advancement of DNA analysis technologies has extended from the field of forensic application to forensic entomology where the ability to retrieve human DNA from insects has implications in terms of both contamination and as a potential source of DNA to be typed [4].

Nevertheless, in some instances, it would be useful as preliminary step to distinguish fly artifacts from genuine bloodstains to determine the dynamics of events [25]. It is worthwhile to consider that crime scenes are not always attended by bloodstain analysis experts, and, however, in some cases, a morphological distinction remains challenging [7, 26], even though a precise frequency of this issue in casework, to the best of our knowledge, has not been reported.

Our study focused to design a DNA-based approach to distinguish fly artifacts from bloodstains.

Forensic genetics laboratories usually perform DNA-based technologies for species-level identification through the mitochondrial genome analysis, generally by sequencing the cytochrome *b* (cyt *b*) region for the versatility of the method and the high PCR efficiency in vertebrates, especially for humans [27, 28]. Even though cyt *b* sequence analysis has been employed for blowflies species identification [29], the design of specific primers to omit human hybridization would have been necessary in our study for the analysis of fly artifacts. Moreover, since in forensic entomology the cytochrome *c* oxidase subunit I and II (COI and COII) genes analysis has been used as standard locus for unambiguous species discrimination as well as evolutionary relationship studies because they have a high rate of genetic variation [30], COI was selected for our molecular assay.

In this study, fly artifacts from *Calliphora vomitoria* feeding on human blood were analyzed, considering that the first arrivers at a carcass are especially blowflies of *Calliphoridae* family [15].

Since Rivers et al. [10–12] has reported that only artifacts derived from alimentary canal of the adult flies reacted positively to the immunoassay test, we have chosen to analyze fly artifacts more likely originated from regurgitation or fecal elimination processes, excluding transfer pattern stains produced by tarsi or other body parts, because fly touch DNA was not expected.

In our study, by using a newly designed primer’s pair to amplify COI gene which contains species-specific single base variations, positive results were obtained for the 94% of fly artifact samples independently from morphology and color type. This success rate is likely due to the amplification of a short fragment of the COI gene that could permit to analyze DNA even at a certain level of degradation not affecting the sequence of interest. The limited number of negative samples (4/68), showing circular or elliptical shape and different color features, does not permit to establish an association with the morphological characteristics. Negative results could be explained by a low quantity of fly biological material, not suitable for DNA amplification. In order to support this hypothesis, a quantification system to determine DNA quantity and quality of fly artifacts would be desirable, also to evaluate the fly DNA target concentration for PCR reaction, based in our study only to stain dimensions. Nevertheless, we cannot also exclude an accidental sampling of stains produced by tarsi.

Regarding the origin of DNA, we suppose that it could derive from fly cells or free DNA in the regurgitated or defecated spots. From this point of view, the reported “bubbling” behavior of flies could represent a source of DNA enrichment by the evaporation of water and concentration of food [4]. However, the modality of exude and reingested fluid by adult flies could lead also to nucleic acid degradation and might explain the failure of COI fragment amplification in our negative samples.

Moreover, given that in previous study the amount of human DNA increased over a time-span of 400 days, suggesting the presence of an inhibitor affecting the extraction DNA process losing its action over time [4], a chronological factor could be implied for negative fly DNA samples collected over 60 days. To evaluate this hypothesis, studies including different sampling times of fly artifacts will be developed.

Our molecular method, following the guidelines, showed high sensitivity, obtaining successful results up to 0.015 ng/ml of DNA target, but we are aware that the quantification of fly DNA needs a more accurate system than the photometric measurement of nucleic acids applied in this study.

Considering that in many regurgitates and in feces the ratio of fly DNA to human DNA may be very low, we tested mixture analyses up to a ratio of 1:100 of fly: human DNAs obtaining a high specificity, and no human DNA contamination was seen by sequencing the PCR products.

The following sequencing and comparison with a sequences database, including the majority of species-specific regions with high similarity scoring, allow the species identification of insect who deposited artifacts, which could correlate with post mortem interval (PMI) and thus help forensic investigations. In our study, the 99–100% identity of fly artifact sequences with many *C. vomitoria* subjects was proved submitting to the same analysis of the reference sample extracted from pupae, and a full alignment was obtained.

Moreover, DNA-based methods can solve the problem of species identification when scientists are not trained in taxonomy, or when the morphology-based identification does not permit easily the distinguishing of closely related sister species [15].

In our study, the DNA extracts from fly artifacts were used also to analyze human DNA profile of the volunteer’s blood, being the DNA quantity exhibited by qPCR in a range suitable for the more sensitive DNA technologies. Indeed, from all of the 10 quantified fly artifacts, full profiles were obtained, matching the human reference sample as reported in previous studies [7].

The implications for forensic science of the human DNA typing in fly artifacts originated from different biological fluids were described and include the identification of crime scenes, victims, and assailants, as well as the possibility to link person to person or person to action [4]. Further research should be addressed to identify COI sequence of fly’s DNA on artifacts produced after feeding on other biological fluids and deposited on other substrates at different sampling times from deposition, considering that artifacts might remain at crime scenes for several months.

Nevertheless, the DNA transfer via insect vector was reported as a potential source of contamination and could mislead the reconstruction of a crime scene. Hence, the preliminary identification of fly’s DNA from fecal and regurgitation-derived artifacts especially for fly artifacts far from the victim is needed, particularly for the activity level interpretation following secondary, tertiary, or higher orders transfers.

## Conclusions

This study is the first to provide a molecular method to detect fly DNA from artifacts deposited by *Calliphora vomitoria* after feeding on blood, which might be considered confirmatory when, in reconstructing crime events, it is essential the distinction from genuine bloodstains. It does not require a high DNA concentration and could permit also the identification of fly’s species through the COI region sequencing by protocols usually applied in forensic genetic laboratories.

## Supplementary Information

Below is the link to the electronic supplementary material.

Supplementary file1 (PDF 1005 KB)

Supplementary file2 (PDF 77 KB)
