# Supplementary material for: A DNA-based method for distinction of fly artifacts from human bloodstains
Source: Int J Legal Med. 2021 Jun 30;135(6):2155–61. doi: 10.1007/s00414-021-02643-7 (PMC8523429; doi:10.1007/s00414-021-02643-7)
Supplement: Supplementary file 1 — Supplementary file1 (PDF 1005 KB) [file 414_2021_2643_MOESM1_ESM.pdf]

A DNA-BASED METHOD FOR DISTINCTION OF FLY ARTIFACTS FROM HUMAN BLOODSTAINS

International Journal of Legal Medicine

Carla Bini[a,*], Arianna Giorgetti[a], Alessandra Iuvaro[a] , Elena Giovannini[a], Denise Gianfreda[a], Guido Pelletti[a], Susi Pelotti[a]

[a] Department of Medical and Surgical Sciences, Section of Legal Medicine, University of Bologna, via Irnerio, 49, 40126, Bologna, Italy

*Corresponding author**: e-mail: carla.bini@unibo.it

Fig. S1

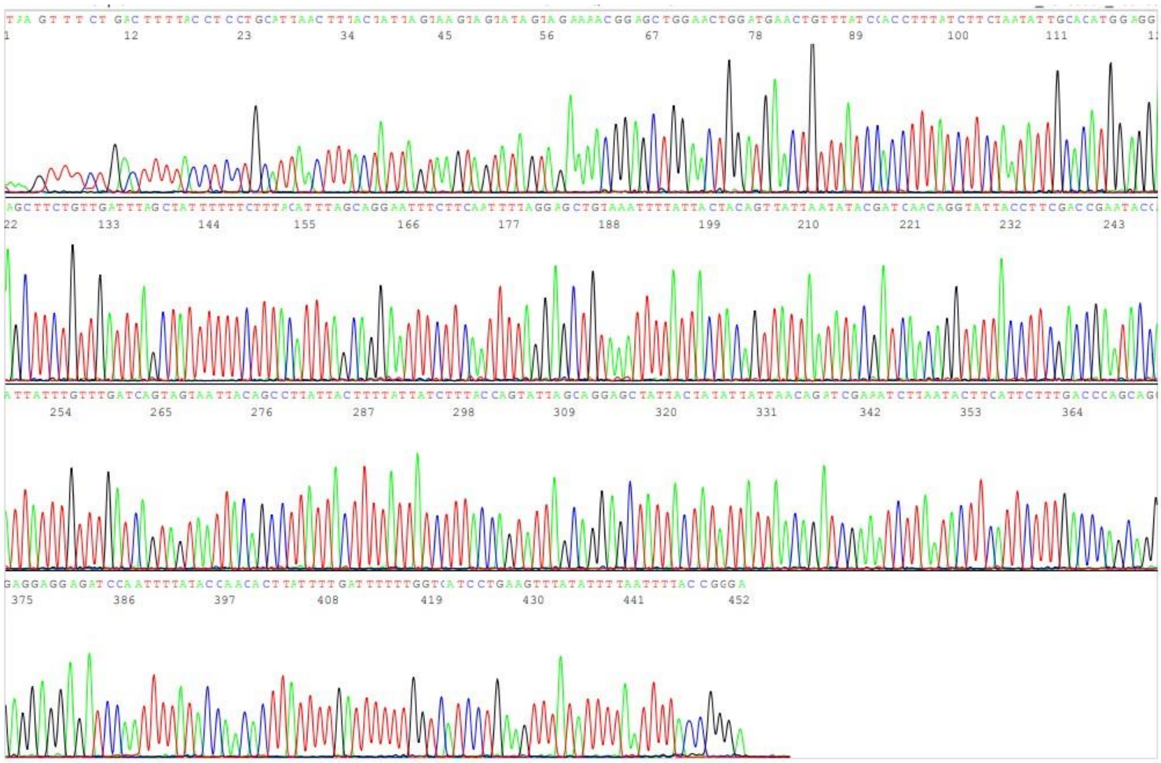

a) Forward sequence of cytochrome *c* oxidase (COI) from *C. vomitoria* pupae

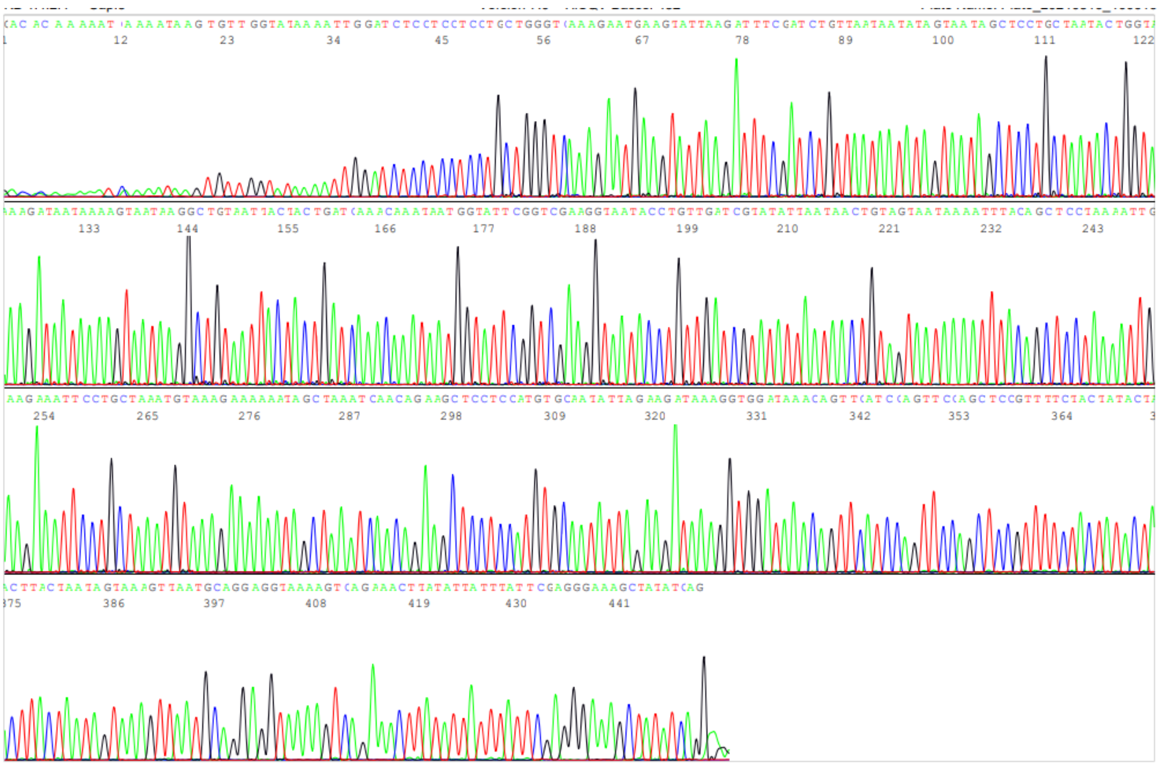

b) Reverse sequence of cytochrome *c* oxidase (COI) from *C. vomitoria* pupae

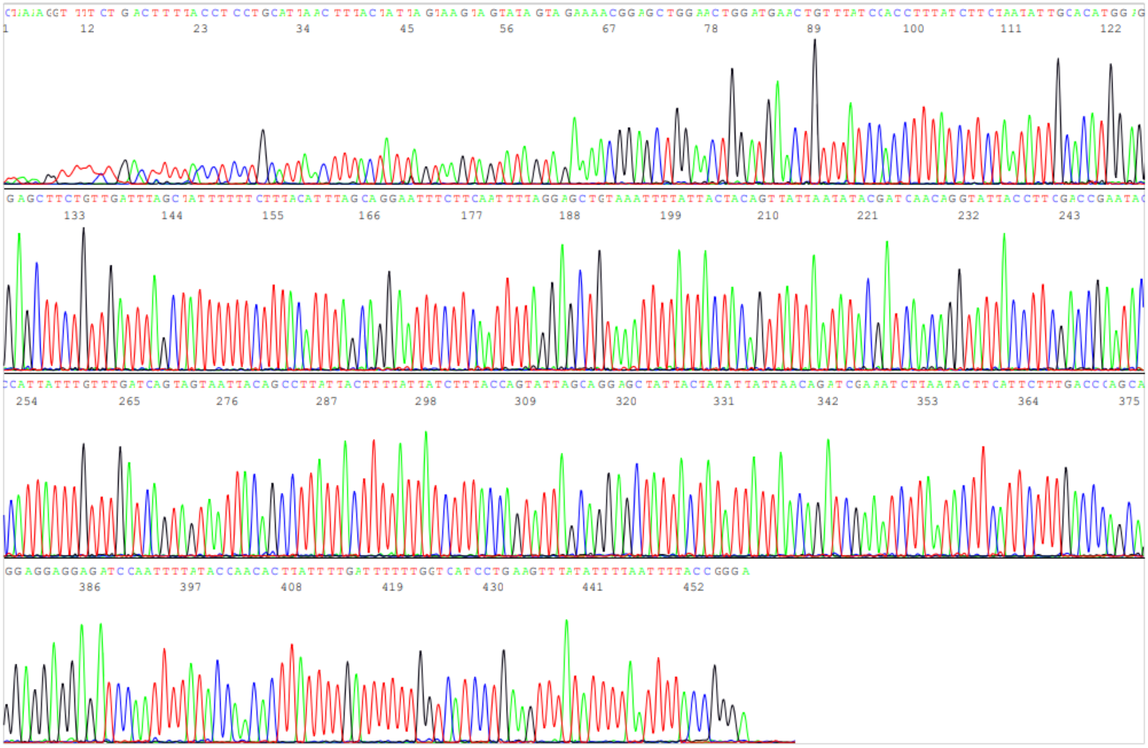

c) Forward sequence of cytochrome c oxidase (COI) from a *C. vomitoria* fly artifact analyzed in our study

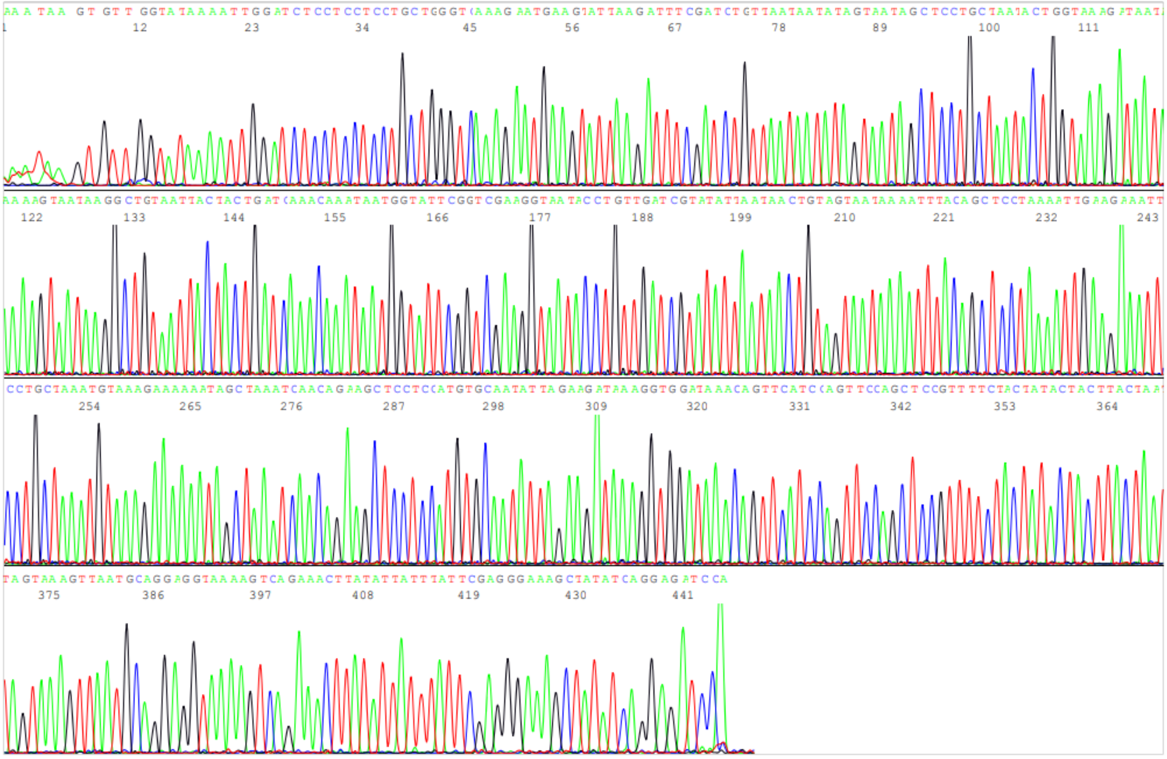

d) Reverse sequence of cytochrome c oxidase (COI) from a *C. vomitoria* fly artifact analyzed in our study
