# Supplementary material for: A DNA-based method for distinction of fly artifacts from human bloodstains
Source: Int J Legal Med. 2021 Jun 30;135(6):2155–61. doi: 10.1007/s00414-021-02643-7 (PMC8523429; doi:10.1007/s00414-021-02643-7)
Supplement: Supplementary file 2 — Supplementary file2 (PDF 77 KB) [file 414_2021_2643_MOESM2_ESM.pdf]

A DNA-BASED METHOD FOR DISTINCTION OF FLY ARTIFACTS FROM HUMAN BLOODSTAINS

International Journal of Legal Medicine

Carla Bini[a,]*, Arianna Giorgetti[a], Alessandra Iuvaro[a] , Elena Giovannini[a], Denise Gianfreda[a], Guido Pelletti[a], Susi Pelotti[a]

[a] Department of Medical and Surgical Sciences, Section of Legal Medicine, University of Bologna, via Irnerio, 49, 40126, Bologna, Italy

*Corresponding author**: e-mail: carla.bini@unibo.it

Fig. S2 Blast alignments of a fly artifact (a) and the pupae reference (b) sequences showing the 99% and 100% identity respectively with *C. vomitoria*

Calliphora vomitoria isolate Vo2 cytochrome oxidase subunit I (COI) gene, partial cds; mitochondrial

Sequence ID: MG969489.1 Length: 1539 Number of Matches: 1

Range 1: 292 to 744 GenBank Graphics ▼ Next Match ▲ Previous Match

Score	Expect	Identities	Gaps	Strand
826 bits(447)	0.0	452/454(99%)	1/454(0%)	Plus/Plus

```
Query  2    ATAAGGTTTCTGACTTTTACCTCCTGCATTAACTTTACTATTAGTAAGTAGTATAGTAGA  61
Sbjct  292  ....-.......................................................  350

Query  62   AAACGGAGCTGGAACTGGATGAACTGTTTATCCACCTTTATCTTCTAATATTGCACATGG  121
Sbjct  351  ............................................................  410

Query  122  AGGAGCTTCTGTTGATTTAGCTATTTTTTCTTTACATTTAGCAGGAATTTCTTCAATTTT  181
Sbjct  411  ............................................................  470

Query  182  AGGAGCTGTAAATTTTATTACTACAGTTATTAATATACGATCAACAGGTATTACCTTCGA  241
Sbjct  471  ............................................................  530

Query  242  CCGAATACCATTATTTGTTTGATCAGTAGTAATTACAGCCTTATTACTTTTATTATCTTT  301
Sbjct  531  ............................................................  590

Query  302  ACCAGTATTAGCAGGAGCTATTACTATATTATTAACAGATCGAAATCTTAATACTTCATT  361
Sbjct  591  ............................................................  650

Query  362  CTTTGACCCAGCAGGAGGAGGAGATCCAATTTTATACCAACACTTATTTTGATTTTTTGG  421
Sbjct  651  ............................................................  710

Query  422  TCATCCTGAAGTTTATATTTTAATTTTACCGGGA  455
Sbjct  711  ..............................T...  744
```

a)

Calliphora vomitoria isolate Vo2 cytochrome oxidase subunit I (COI) gene, partial cds; mitochondrial

Sequence ID: MG969489.1 Length: 1539 Number of Matches: 1

Range 1: 291 to 736 GenBank Graphics ▼ Next Match ▲ Previous Match

Score	Expect	Identities	Gaps	Strand
824 bits(446)	0.0	446/446(100%)	0/446(0%)	Plus/Plus

```
Query  1    TATAAGTTTCTGACTTTTACCTCCTGCATTAACTTTACTATTAGTAAGTAGTATAGTAGA  60
Sbjct  291  ............................................................  350

Query  61   AAACGGAGCTGGAACTGGATGAACTGTTTATCCACCTTTATCTTCTAATATTGCACATGG  120
Sbjct  351  ............................................................  410

Query  121  AGGAGCTTCTGTTGATTTAGCTATTTTTTCTTTACATTTAGCAGGAATTTCTTCAATTTT  180
Sbjct  411  ............................................................  470

Query  181  AGGAGCTGTAAATTTTATTACTACAGTTATTAATATACGATCAACAGGTATTACCTTCGA  240
Sbjct  471  ............................................................  530

Query  241  CCGAATACCATTATTTGTTTGATCAGTAGTAATTACAGCCTTATTACTTTTATTATCTTT  300
Sbjct  531  ............................................................  590

Query  301  ACCAGTATTAGCAGGAGCTATTACTATATTATTAACAGATCGAAATCTTAATACTTCATT  360
Sbjct  591  ............................................................  650

Query  361  CTTTGACCCAGCAGGAGGAGGAGATCCAATTTTATACCAACACTTATTTTGATTTTTTGG  420
Sbjct  651  ............................................................  710

Query  421  TCATCCTGAAGTTTATATTTTAATTT  446
Sbjct  711  ..........................  736
```

b)
